# Non-Traditional Non-Immunological Risk Factors for Kidney Allograft Loss—Opinion

**DOI:** 10.3390/jcm12062364

**Published:** 2023-03-18

**Authors:** Titus Andrian, Lucian Siriteanu, Andreea Simona Covic, Cristina Alexandra Ipate, Adelina Miron, Corneliu Morosanu, Irina-Draga Caruntu, Adrian Covic

**Affiliations:** 1 Nephrology Clinic, Dialysis and Renal Transplant Center, C. I. Parhon University Hospital, 700503 Iasi, Romania; titus.andrian@umfiasi.ro (T.A.);; 2 Department of Internal Medicine, ‘Grigore T. Popa’ University of Medicine, 700115 Iasi, Romania

**Keywords:** allograft loss, risk factors, kidney transplant, long-term survival, chronic allograft nephropathy, non-traditional

## Abstract

Rates of late allograft loss have improved slowly in the last decades. Well described traditional risk factors that influence allograft survival include cardiovascular events, rejection, infections and post-transplant neoplasia. Here, we critically evaluate the influence of several non-immunological, non-traditional risk factors and describe their impact on allograft survival and cardiovascular health of kidney transplant recipients. We assessed the following risk factors: arterial stiffness, persistent arteriovenous access, mineral bone disease, immunosuppressive drugs residual levels variability, hypomagnesemia, glomerular pathological alterations not included in Banff criteria, persistent inflammation and metabolic acidosis.

## 1. Introduction

Recent high-level evidence confirms the superiority of transplantation in reducing all-cause mortality for most patients with end-stage kidney disease [1]. In the last decades, significant advances in the care of transplanted patients have led to massively improved 1 year graft survival [2]. However, long-term survival only slightly recorded improvement, highlighting the paramount importance of individualizing risk factors that alter the allograft health [3]. Traditionally, progressive allograft dysfunction is mainly mediated by well-described clinical entities, such as marginal donor, rejection, non-compliance, calcineurin-inhibitors nephrotoxicity, recurrent glomerulonephritis and viral nephropathies [4]. Transplanted patients experiencing transition to dialysis after a failed allograft are exposed to several negative-impact outcomes: high burden of distress and comorbidities, increased risk of hospitalizations, excess mortality and higher economic cost [5]. Thus, addressing the risk factors associated to allograft loss may extend survival and offer better outcomes as patients returning to dialysis lose the benefits of transplantation [6]. Here, we conducted a literature search in order to describe other “non-traditional” mediators of kidney allograft loss, evaluating their impact on survival outcomes.

## 2. Search Strategy and Inclusion Criteria

We searched three databases: Medline, EMBASE and Cochrane Library, and included studies from January 2000 to December 2022 with the following terms: “kidney graft survival”, “kidney graft loss”, “failed kidney transplant”, “chronic allograft nephropathy”, “risk factors”, “chronic kidney disease progression”.

## 3. Causes of Long-Term Allograft Dysfunction and Allograft Loss

Late allograft failure is a heterogenous background picture of several multifactorial pathogenic entities that all contribute to kidney dysfunction in transplant patients. Naesens et al. elegantly described data derived from failing grafts: Importantly, one quarter of biopsies exhibit more than one coexisting disease, and almost one third of patients with lost allograft had no specific diagnosis on the last biopsy performed but highlighted the important prognostic capacity of the extent of chronic damage [7]. Consequently, given the impact of the cumulative active and chronic injuries insulting the allograft, even histology may fail to underline one specific offending disease process. Causes that lead to loss of graft function include immunologic and non-immunologic mechanisms (Table 1) [8].

Recent efforts have focused on better characterization of allograft loss and described a more complex picture of the multifactorial pathogenic processes. A study conducted by Mayrdorfer et al. examining 303 death-censored graft losses among 1642 transplant recipients discriminated primary failure causes (responsible for more than 50% persistent estimated glomerular filtration rate decrease) from secondary failure causes (contribution to less than 50% estimated glomerular filtration rate decline) and analyzed data in a time-dependent manner. Late allograft failure was mainly attributed to intercurrent medical events, defined as cardiovascular events or infections conducting to prominent decline in renal function, and representing almost 40% of late graft failures. Moreover, antibody-mediated rejection met increasing frequencies with time as a primary failure cause (48.6% of graft failures at more than 10 years from transplant intervention) while T-cell mediated rejection had a sharp decline with transplant vintage. Interestingly, calcineurin-inhibitors toxicity was isolated in only two cases as a primary cause, but it was described as the most frequent secondary cause responsible for graft loss (64 of 240 late graft failures). The authors once again highlighted the multifactorial etiology of transplant failure [9].

## 4. Prediction Systems for Allograft Survival

The ability to predict the evolution of the transplanted kidney is of great significance and has been the center of some valuable research. Search of prognostic factors begins even before implantation with evaluation of donor quality. Preimplantation histology may provide important clues but clinical decisions of implanting or discarding the kidney based on this data have shown conflicting results. In a recent large multinational study, discarded kidneys due to biopsy alterations could have been offered to patients with improved survival and quality of life. Data shows 70% of implanted similar grafts matched with the refused grafts due to biopsy were functioning at 10 years [10]. A multi-center randomized study involving all adult kidney transplant centers in the United Kingdom will assess the impact of urgent preimplantation biopsies in selection of kidneys and will evaluate the outcome of transplanted patients [11].

Prediction models can be computed by means of artificial intelligence and deep learning models to predict evolution of estimated glomerular filtration rate after kidney transplantation. Raynaud et al. identified, after performing multinomial regression models, seven significant determinants of progression: donor age, estimated glomerular filtration rate, proteinuria, graft scarring, graft interstitial inflammation and tubulitis, microcirculation inflammation and circulating anti-HLA donor specific antibodies. Resulting computed trajectories were significantly associated with progression to allograft loss [12].

Other techniques proved good forecast capability utilizing sequence-to-sequence modelling in order to create a machine learning algorithm-based prediction model that is based on inputting repeated estimated glomerular filtration rate measurements [13].

Recently, an interesting dynamic prediction tool that incorporates histological, immunological and clinical parameters together with repeated measurements of graft function was validated in multiple cohorts and proved superior prediction performance of allograft survival compared to other similar systems. Individualized parameters that altered survival were estimated glomerular filtration rate, proteinuria, delay between transplant and evaluation, Banff categories (glomerulopathy + peritubular capilaritis, interstitial fibrosis with tubular atrophy—IFTA, inflammation + tubulitis, chronic glomerulopathy), mean fluorescence intensity of anti-HLA donor specific antibodies and repeated measurements of eGFR. The iBox system has proved good prediction ability of graft survival and it has been validated as a valuable endpoint in clinical trials in transplantation [14,15].

## 5. Non-Traditional Risk Factors for Allograft Dysfunction

### Non-Traditional Cardiovascular Risk Factors (Arterial Stiffness)

**Figure 1:**
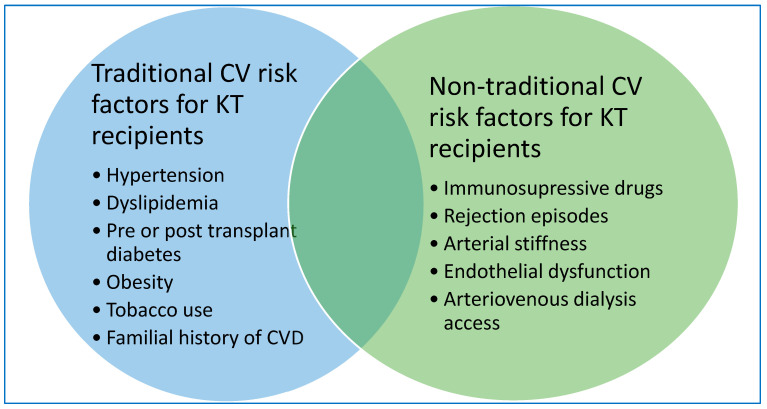
Traditional and non-traditional CV risk factors in KT recipients.

Cardiovascular disease has recently gained well-deserved attention since it represents the main driver of comorbidity and mortality in kidney transplant recipients. Pregnant emphasis was attributed to defining and addressing traditional cardiovascular risk factors in improving survival outcomes in this population. Management of these associated relevant comorbidities have been reviewed elsewhere and they do not meet the purpose of this paper (Figure 1) [16,17].

The association between increased values of arterial stiffness and eGFR decline has been noted in the general chronic kidney disease (CKD) population and suggests that the resulting hemodynamic stress insults the kidney by means of endothelial dysfunction and microvascular ischemia. Chronic inflammation, oxidative stress and overactivation of the renin–angiotensin systems may represent other possible mechanistic underpinnings [18]. Aortic pulse–wave–velocity as a proxy and measure of vascular stiffness is higher in kidney transplant recipients compared to healthy subjects [19]. Additionally, aortic stiffness determined by the same method is associated with adverse outcomes in graft recipients, including altered allograft function [20], increased cardiovascular events [21] and higher mortality [22]. Although successful transplantation tends to lower arterial stiffness, excessive vascular damage still subsists probably as an irreversible result of previous uremic milieu [23]. Several plausible de-stiffening strategies have been employed in CKD populations but were not adequately studied in renal transplant patients. Among them, endothelin receptor antagonists may show promise. Moreover, vascular health in transplanted patients is heavily influenced by associated immunosuppressive drugs. Corticosteroids damage microvasculature by hydrosaline retention and renin–angiotensin–aldosterone system activation. Cyclosporine leads to increased stiffness, and thus, a switch to tacrolimus may prove beneficial. Belatacept is postulated to reduce vascular inflammation, but future, more potent studies should clarify this plausible impact [24,25,26].

## 6. Persistent Arteriovenous Fistula

Continuous functioning arteriovenous fistula after kidney transplantation has the potential to worsen the maladaptive remodeling changes in the cardiovascular system and alter kidney function. A meta-analysis addressing the impact of ligating the arteriovenous fistula showed that patients undergoing access occlusion had better cardiac morphological parameters and better kidney allograft function [27].

Furthermore, patency at 5 years was only 61% and arteriovenous fistula-related complications were frequently reaching about 30% of patients. On the other hand, preservation of the vascular access may provide the advantage of an optimal backup strategy if transition to dialysis is needed. Consequently, decisions should be individualized on several parameters, mainly based on renal and cardiac functions and on selecting complications-prone profile of patients [28].

## 7. Mineral and Bone Disease after Kidney Transplant and Impact on Outcomes

After kidney transplantation, particularly older patients find themselves at an increased risk for pathological fractures. The fractures appear multifactorial in nature (previous bone disease, frailty, immunosuppressive regimens and osteoporosis) and are associated with unfavorable outcomes, such as subsequent graft loss or mortality. It remains unclear whether only the high end of mineral bone disease spectrum (fractures) is linked to allograft survival decline or if other biologic entities contribute as well [29].

Persistent mineral abnormalities after kidney transplantation mainly include hypercalcemia and hypophosphatemia. Previous severe hyperparathyroidism and high levels of circulating PTH and FGF-23 are established risk factors for altered mineral metabolism post-transplantation. However, impact on outcomes is unclear. As they may not contribute directly to allograft loss and rapid eGFR decline, they are the main drivers of vascular calcifications affecting graft vessels and, interestingly, of tubulointerstitial calcifications on allograft biopsies [30,31].

Cinacalcet has proven its ability to correct persistent hyperparathyroidism after kidney transplantation and the associated metabolic derangements but without impact on bone mineral density or allograft survival [32]. Data from Cruzado et al. elegantly shows in a randomized study that parathyroidectomy compared to cinacalcet offers improved correction of hyperparathyroidism and hypercalcemia but fails to prove a difference on hard outcomes such as eGFR decline or calcification, probably because of a small sample size and insufficient long-term follow-up [33].

Biphosphonate therapy in kidney transplant recipients has the clear benefit of improving bone mineral density. Recent studies have proven low rates of adynamic bone disease, strengthening the safety profile of this medication. However, there is no clear data on rates of fractures and there are no benefits or harms related to renal function evolution in kidney transplant recipients [34].

Assessment of pre-transplantation profile of mineral bone disease phenotype may predict survival and high-risk patients. In a cohort of more than 11,000 patients, recipients that exhibited higher levels of pre-transplant serum alkaline phosphatase (>120/U/L) were subject to increased post-transplant mortality. No association was found between circulating PTH or calcium and post-transplant evolution [35].

The negative correlation between graft survival and vitamin D deficiency has been described in an observational manner [36]. Insufficient levels of vitamin D are postulated to interfere with transplant tolerance and may lead to increased risk of infections and neoplasia [34]. No adequately powered trials have yet been published on this matter and correcting vitamin D deficiency is not recommended in order to improve graft survival. Vitamin D receptor agonist agents showed promise as they illustrated anti-proteinuric effects in kidney transplant recipients [37]. A study described the effect of paricalcitol that lowered PTH levels but failed to reduce proteinuria and had no other effect on eGFR or arterial health [38]. According to KDIGO guidelines, vitamin D should be supplemented according to circulating levels as in the general population [39].

Current therapeutic armamentarium provides means to effectively correct mineral abnormalities in kidney transplant recipients. However, impact of described treatments on graft survival have been insufficiently studied and proved no significant benefits.

## 8. Immunosuppressive Drug Variability

Excessive immunosuppression contributes to significant complications linked to allograft loss; infections, malignancy and polyoma virus nephropathy account for about one third of graft losses [9]. Because of a narrow therapeutic window and potential side effects, immunosuppressive drugs need to be followed and adjusted delicately on the base of their blood residual concentrations. Despite stable doses, some patients experience wide variability between subtherapeutic and supratherapeutic drug levels [40]. This fluctuation may be explained primarily by non-adherence, but other factors contribute as well, including alimentation, epigenetic profile, drug-interactions. High variability of tacrolimus and cyclosporine levels predicts significant poorer outcomes as suggested by the results of multiple studies that show the significant interaction with late allograft rejection or graft loss [41,42,43,44].

Interestingly, another tool available for the evaluation of drug exposure is the time in therapeutic range—addresses the interval of time that a patient is exposed to correct predefined range of values of immunosuppression. Patients with high variability and time in therapeutic range < 40% were at increased risk of de novo donor-specific antibodies and at an estimated four times greater risk of graft loss by 5 years [45].

## 9. Hypomagnesemia

Hypomagnesemia represents one of the most frequent electrolyte disturbances post-transplantation [46]. Several specific factors contribute to magnesium waste: urinary-losses induced by calcineurin inhibitors, mTOR inhibitors or diuretics; metabolic acidosis; volume expansion with intravenous fluids; decreased gastro–intestinal absorption due to diarrhea or proton pump inhibitors [47].

Magnesium deficit exhibits diabetogenic effects and it represents a proven independent risk factor for post-transplant diabetes mellitus [48,49]. Recent data also note the increased risk of Mg depleted patients to develop urinary tract infections and viral infections [50].

Reports on mortality are conflicting and derived from observational retrospective studies. There have been signals of the possible interaction between hypomagnesemia and graft dysfunction [51]. Recently, a large cohort study proved a U-shaped association between serum magnesium and mortality in kidney transplant recipients. Furthermore, deregulated magnesium metabolism had an impact on cardiovascular mortality [52]. On the other hand, hypomagnesemia has been associated with a better short-term and long-term patient and allograft survival. It has been hypothesized that magnesium depletion may only represent a marker of better tubular graft function [53,54].

Magnesium metabolism, however, is important and may have implications in long-term outcomes as it interferes with glycemic alterations, endothelial dysfunction and cardiovascular abnormalities. In addition, correction is cumbersome and magnesium levels may not reflect true stores. No significant trials regarding supplementation and the associated impact on graft function have been conducted [55].

Overall, due to conflicting results and the presence of many confounders, such as the use of CNI, nutritional status, measurement issues, other additional studies addressing this problem will evaluate true impact.

## 10. Other Histologic Features—Beyond Banff Criteria

The kidney transplant biopsy interpretation is submitted to a well described and standardized score system—Banff classification. Diagnostic entities are established by pathologists after grading several acute and chronic lesions [56]. However, evolving data offer new histopathological markers relevant to graft outcomes that are not yet covered by the Banff system.

Denic et al. conducted a study with repeated biopsies in well-functioning transplant recipients at 5 years post-implantation. Three parameters previously linked to unfavorable graft outcomes were examined: glomerular volume, global glomerulosclerosis and ischemic appearance of glomeruli. All three parameters combine successfully predicted death-censored graft failure at 5 years. Moreover, glomerular ischemia is significantly associated with allograft loss, even after adjustment for Banff scores [57].

Moreover, other recent data highlight the impact of early subclinical inflammation detected on surveillance biopsies on allograft survival. Patients with subclinical inflammation and tubulitis were exposed to a higher hazard of acute rejection and death-censored allograft loss [58].

## 11. Persistent Inflammation

Implantation of the kidney allograft is often realized in uremic patients exhibiting a state of inflammatory milieu. Moreover, inflammation is enhanced by perioperative events and induced by other stressors. For example, brain death is linked to increased cytokine excretion and ischemia-reperfusion injury can lead to delayed graft function. All forms of rejection are responsible for elevated markers of inflammation, and viral infections could be a source of enhanced cytokine production leading to overreactive innate immunity [59]. Atherosclerosis is now considered a disease centered on disrupted inflammation triggered by lipid accumulation in arterial walls. Elevated levels of inflammatory biomarkers are associated with increased cardiovascular risk in kidney transplant recipients [60]. Inflammation may also lead to a prothrombotic state, endothelial dysfunction and platelet activation [61].

The persistent inflammatory state is multifactorial and contributes to graft dysfunction by several mechanisms: triggering chronic hypoxia, extracellular matrix deposition and extending graft fibrosis [59]. Furthermore, persistent infiltration with inflammatory cells in scarred areas of fibrosis on biopsy is one of the main predictors of allograft dysfunction [62].

## 12. Metabolic Acidosis

Metabolic acidosis identifies as one of the most common complications in kidney transplant recipients. The fall of bicarbonate and pH levels with a decrease in serum partial pressure of carbonic dioxide occurs mainly when the glomerular filtration rate falls under 30 mL/min in patients with chronic kidney disease. This threshold is higher in kidney transplant recipients, showing that metabolic acidosis may occur at higher GFRs in these patients [63,64]. The overall reported prevalence varies from 11% to over 50% [65]. The pathophysiological mechanisms seem to be particular to the kidney transplant status and act especially in the early post-transplant period. Metabolic acidosis is mostly caused by immunosuppressive therapy, which usually includes a calcineurin inhibitor, suboptimal capacity of angiogenesis due to the tubulointerstitial lesions caused by ischemia and allograft rejection. Nevertheless, donation and donor-associated factors such as ischemia time, donor type, age, sex and kidney function are to be considered [66,67,68].

Data on allograft survival are pointing to metabolic acidosis as a risk factor for graft dysfunction in the long term. Park et al. conducted a study that showed that low TCO2 concentrations 3 months after transplant are associated with an increased risk of graft loss and death-censored graft failure [67]. Furthermore, metabolic acidosis increased the risk of mortality [69]. Moreover, the severity of metabolic acidosis may be associated with progressive allograft dysfunction [67].

Thus, these associations require the application of therapeutic measures to correct acidosis. From this point of view, the data are limited, with most studies focusing on alkali therapy. If supplementation with sodium bicarbonate is effective in patients witch chronic kidney disease, the data in kidney transplant recipients are limited. Schulte at al. evaluated the effect of sodium bicarbonate in kidney transplant recipients with chronic metabolic acidosis. Treatment with sodium bicarbonate was associated with an increased risk of graft failure with no changes in mortality. Authors speculated that the interference with gastric acid pH of oral bicarbonate intake may lead to altered absorption of mycophenolate mofetil. Consequently, patients may be exposed to lower plasma concentrations of mycophenolic acid [69]. The recent randomized Preserve–Transplant Study failed to show the benefit of correcting metabolic acidosis with sodium bicarbonate in the attempt to slow the decline of estimated GFR [70].

## 13. Summary

**Figure 2:**
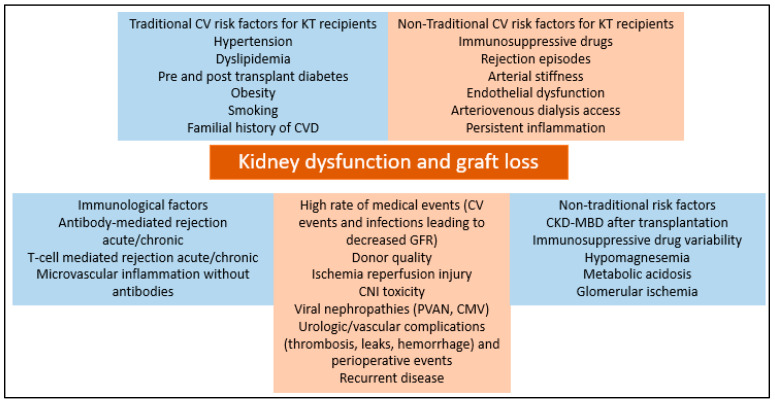
Risk factors for kidney dysfunction and allograft loss in kidney transplant recipients.

Long-term survival of kidney allografts is altered mainly by cardiovascular disease, malignancy and infections. However, in order to maximize survival, other relevant pathogenic entities need to be addressed. Care of kidney transplant patients is complex and needs to be centered also on many potential risk factors that can influence long-term survival, consisting of the following: metabolic derangements such as hypomagnesemia, metabolic acidosis and mineral bone disease after transplantation, cardiovascular disease manifesting as arterial stiffness, immunosuppressive drug metabolism and non-adherence, persistent inflammation and glomerular changes not highlighted by Banff criteria. (Figure 2).

## Figures and Tables

**Table 1:** Traditional immunologic and non-immunologic risk factors for allograft loss (Adapted from ref. [8,9]).

Immunological Factors	Non-Immunological Factors
Antibody-mediated rejection: acute/chronic T-cell mediated rejection: acute/chronic Microvascular inflammation without antibodies	Medical events (cardiovascular events and infections leading to decreased estimated glomerular filtration rate—eGFR) Donor quality: age, comorbidities Ischemia reperfusion injury Calcineurin inhibitors—CNI toxicity Viral nephropathies (polyoma virus associated nephropathy—PVAN, cytomegalovirus—CMV) Urologic/vascular complications (thrombosis, stenosis, leaks, hemorrhage) and perioperative events Recurrent disease
